# A Pancreatic Inflammatory Myofibroblastic Tumor with Spontaneous Remission: A Case Report with a Literature Review

**DOI:** 10.3390/diagnostics9040150

**Published:** 2019-10-17

**Authors:** Hiroyuki Matsubayashi, Katsuhiko Uesaka, Keiko Sasaki, Seitaro Shimada, Kazunori Takada, Hirotoshi Ishiwatari, Hiroyuki Ono

**Affiliations:** 1 Division of Endoscopy, Shizuoka Cancer Center, Shizuoka 411-8777, Japan; s.shimada@scchr.jp (S.S.); ka.takada@scchr.jp (K.T.); h.ishiwatari@scchr.jp (H.I.); h.ono@scchr.jp (H.O.); 2 Division of Hepato-pancreaticobiliary Surgery, Shizuoka Cancer Center, Shizuoka 411-8777, Japan; k.uesaka@scchr.jp; 3 Division of Pathology, Shizuoka Cancer Center, Shizuoka 411-8777, Japan; k.sasaki@scchr.jp

**Keywords:** pancreas, inflammatory myofibroblastic tumor, spontaneous regression, diagnosis

## Abstract

The inflammatory myofibroblastic tumor (IMT) is a rare tumor that can develop in any systemic organ. Its features are generally benign, but it often resembles malignancies and is treated surgically. Our patient was an 82-year-old female complaining of abdominal discomfort. Computed tomography demonstrated a 5 cm, ill-enhanced mass at the pancreas head. Upper gastrointestinal endoscopy revealed a duodenal submucosal tumor with apical erosion. Endoscopic ultrasonography (EUS) demonstrated a heterogeneous, low-echoic pancreas mass without clear margins. Fine-needle aspiration biopsy (FNAB) demonstrated spindle myofibroblastic tissues with lymphoplasmacyte and eosinophil infiltration, confirming an IMT diagnosis. Surprisingly, the tumor spontaneously regressed in one month without medication. Histological diagnosis using EUS-FNAB is essential for the rare pancreatic solid tumor like IMT.

## 1. Introduction

The inflammatory myofibroblastic tumor (IMT) is a rare tumorous lesion that can develop in any systemic organ. It has a relatively young onset (mainly in newborns to young adults) and consists histologically of proliferative myofibroblastic tissues with heavy infiltration of inflammatory cells, mainly lymphocytes and plasma cells [1]. It often shows benign biological behaviors, but it is sometimes accompanied by somatic mutations in clinically important genes [2,3,4,5] that can cause metastasis and/or recurrence [2,5]. The differential diagnosis from malignancies is therefore difficult, especially in high-aged cases, and most IMTs are surgically resected before finally being diagnosed [2,5,6,7,8,9,10,11,12,13,14,15,16,17,18,19,20,21,22,23,24,25,26].

Anti-inflammatory agents, such as corticosteroids and non-steroidal anti-inflammatory drugs (NSAIDS), are effective for some IMTs [27,28], and a small proportion of IMTs regress spontaneously [27,28,29,30,31,32,33,34,35,36,37,38]. To date, several cases of pancreatic IMTs have been reported [6,7,8,9,10,11,12,13,14,15,16,17,18,19,20,21,22,23,24,25,26], but histologically proven cases (proven for myofibroblastic component) with spontaneous remission have hardly been reported.

## 2. Case Presentation

**Figure 1:**
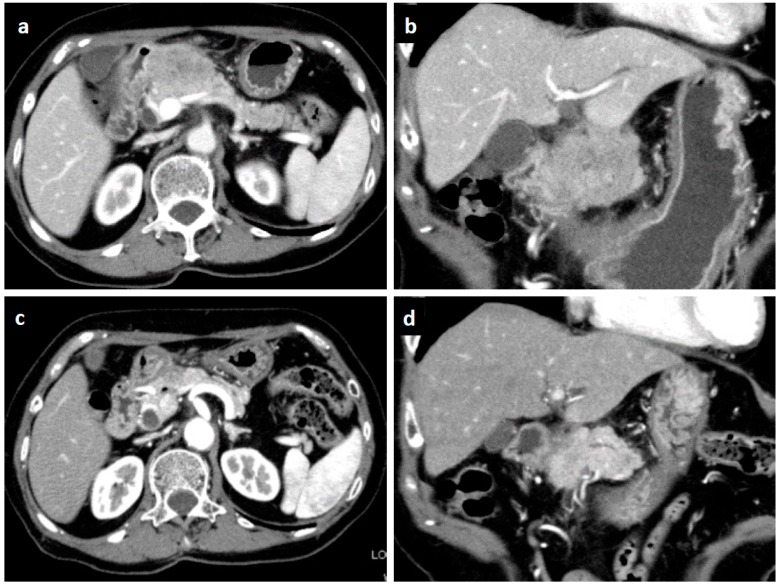
Enhanced computed tomography. An irregular-margined, low-attenuated mass 5 cm in size was seen at the pancreas head (horizontal view (**a**), coronal view (**b**)). One month after the histological diagnosis, the pancreatic mass was markedly shrunken spontaneously (horizontal view (**c**), coronal view (**d**)).

**Figure 2:**
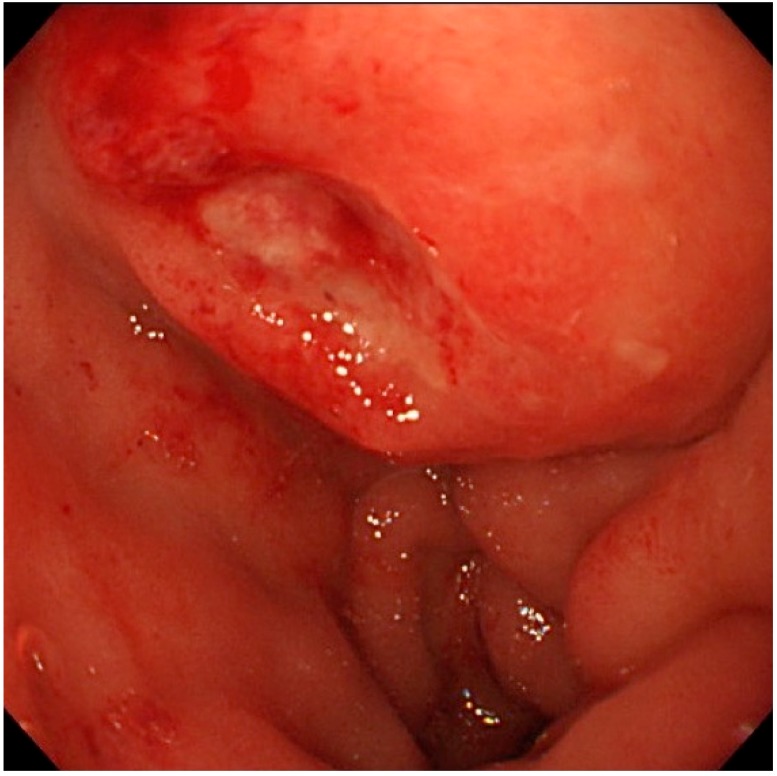
Endoscopic view of the duodenal bulbs. A hemispheric submucosal tumor with apical erosion was recognized.

**Figure 3:**
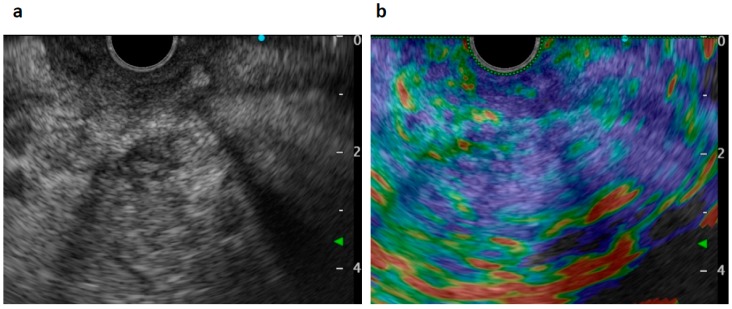
Endoscopic ultrasonography (EUS) views. A heterogeneous low-echoic mass was seen by scanning from the duodenal bulbs (**a**). Elastography showed a heterogeneously hard mass lesion at the pancreas head (**b**).

**Figure 4:**
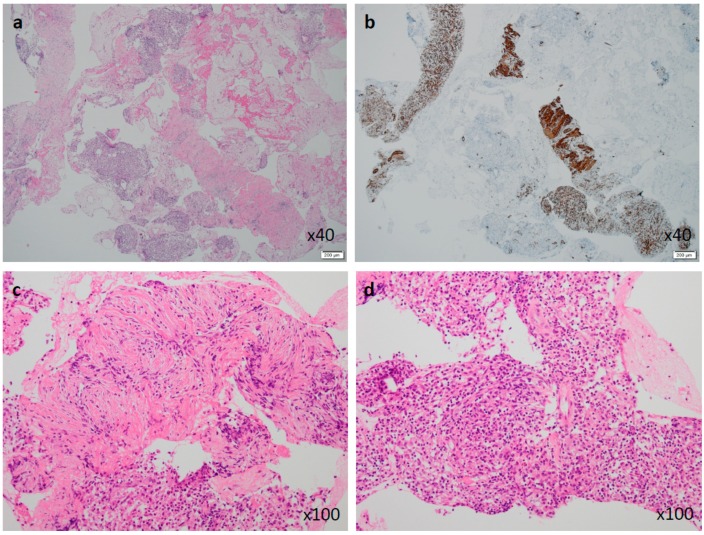
Tissues obtained by EUS-guided fine needle aspiration biopsy (EUS-FNAB). Low-power view of hematoxylin-eosin (HE) staining showed mixed components of dense myofibroblastic tissues and aggregated inflammatory cells (×40). (**a**) Anti-smooth muscle actin was diffusely positive in the myofibroblastic components (×40). (**b**) High-power views showed a myofibroblastic cell component (**c**) and an inflammatory cell component (**d**) without malignant cells (HE, ×100).

An 82-year-old Japanese female was referred to our hospital after a month of complaints of upper abdominal discomfort. First, she visited the nearest hospital and underwent upper gastrointestinal endoscopy that pointed multiple erosions and an extrinsic compression at the posterior pylorus. Medication had been initiated using nizatidine, rebamipide and oxetacaine, but it was not effective. She had a history of hypertension, but her family history was unremarkable. Blood tests showed modestly elevated IgG (1950 mg/dL, normal range: 870–1700 mg/dL) and C-reactive protein (0.43 mg/dL, normal range: ≤ 0.3 mg/dL) but normal readings for other factors, including serum tumor markers (carcinoembryonic antigen, carbohydrate antigen, and soluble IL-2 receptor), HbA1c, and IgG4 (66.1 mg/dL, normal: 4.5–117 mg/dL). Enhanced computed tomography (CT) demonstrated an ill-enhanced mass, 5 cm in size but with unclear margins, located at the pancreas head (Figure 1a,b). Upper gastrointestinal endoscopy revealed a submucosal tumor (SMT) with an apical erosion approximately 1.5 cm in size at the duodenal bulbs (Figure 2). Several faintly enlarged lymph nodes were seen around the pancreas head, but no nodules suggestive of metastasis were visible in the liver or the lungs. Endoscopic ultrasonography (EUS) demonstrated a heterogeneous, low-echoic mass at the pancreas head and body, but no adhesion to the common bile duct. EUS elastography revealed a hardness of the pancreas lesion (Figure 3). Forceps biopsy (Radial Jaw™4, Boston Scientific Japan, 2.2 mm, Tokyo, Japan) from the duodenal SMT was not informative, but EUS-guided fine needle aspiration biopsy (FNAB) showed abundant spindle myofibroblast tissues with eosinophilic and lymphoplasmacytic cell infiltration (Figure 4). FNAB was performed with two punctures from the duodenal bulbs, with each puncture performed with 10 strokes using a 22-gauge Franseen-tip needle (Acquire™, Boston Scientific Japan) with 10 mL of negative pressure. No malignant cells were seen. The spindle cells were positive for anti-smooth muscle antibody (ASMA) and desmin but negative for discovered on GIST-1 (DOG-1), c-Kit, CD34, S-100, and ALK. Only six IgG4-positive cells were recognized in high-powered views, and no obliterative phlebitis or storiform fibrosis was detected. These findings led to the diagnosis of IMT.

**Figure 5:**
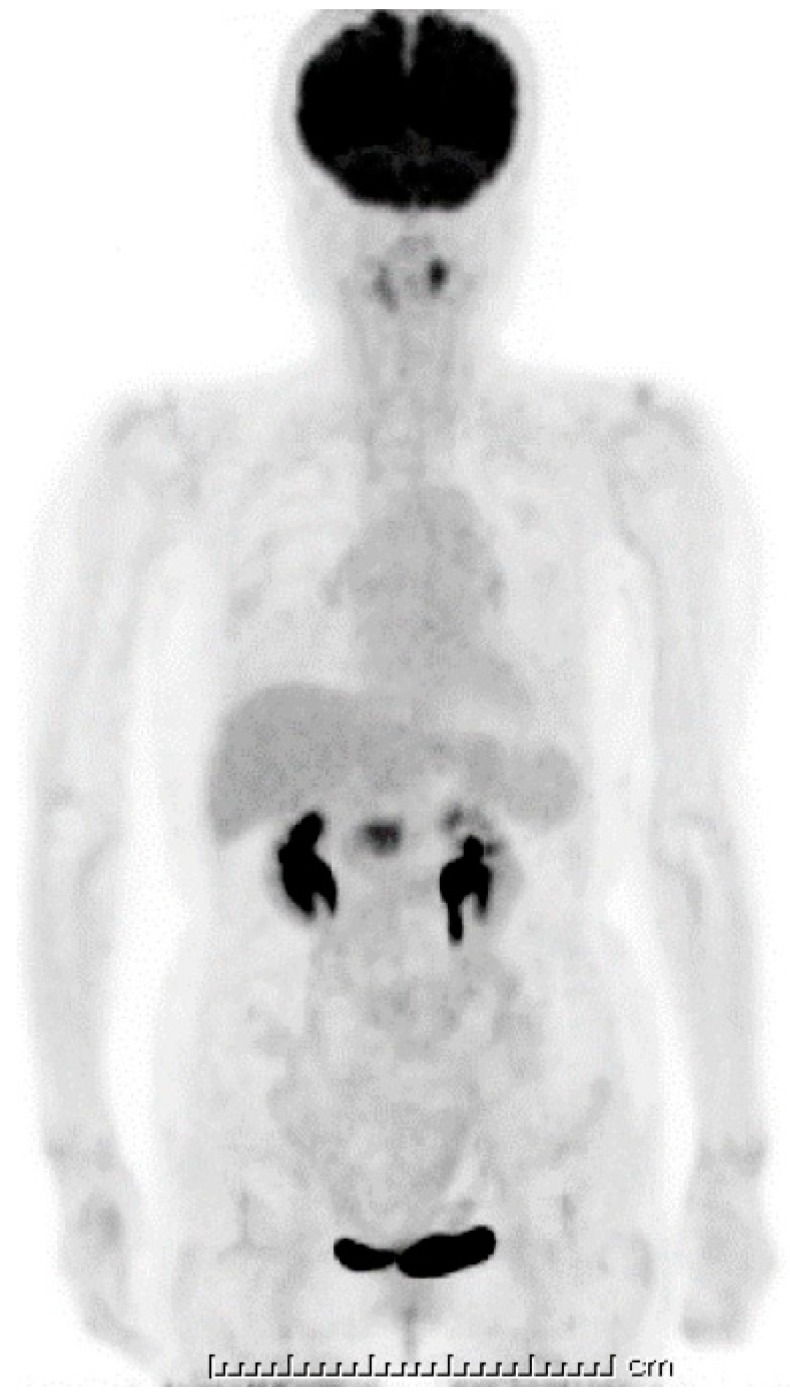
^18^F-fluorodeoxyglucose positron emission tomography (FDG-PET). A strong uptake of FDG was visible at the pancreas head (SUVmax: 6.95); however, this looked smaller when compared with the initial computed tomography (CT) image. The one unit of under bar is indicating 1cm and total bar length is 25 cm.

**Figure 6:**
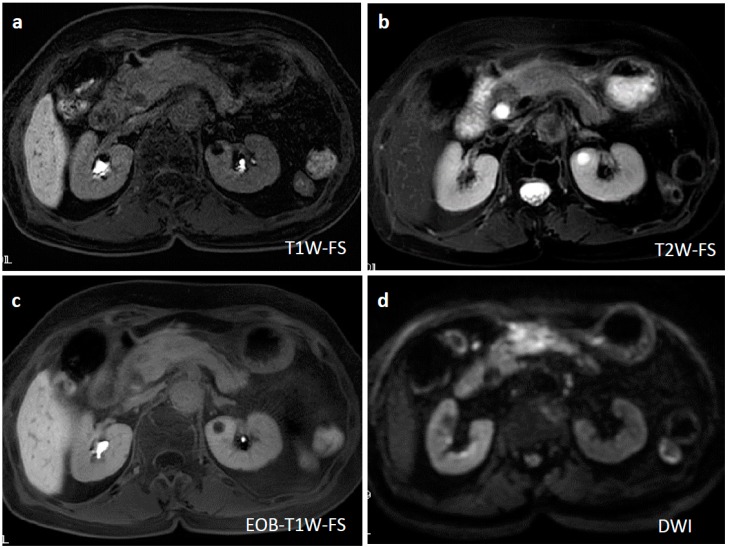
Magnetic resonance imaging (MRI) at two weeks after EUS-FNAB and three weeks after the initial CT. A T1-weighted MRI showed an iso-intensity signal (**a**), a T2-weighted image showed a faint low-intensity signal (**b**), a gadoxetate sodium-injected MRI demonstrated a slightly weak enhancement (**c**), and a diffusion-weighted MRI revealed heterogeneously repressed diffusion ability at the pancreatic lesion (**d**).

Ten days after FNAB, positron emission tomography showed abnormal ^18^F-fluorodeoxyglucose uptake (SUVmax: 6.95); however, the pancreatic lesion seemed to have shrunk to 2.5 cm in size (Figure 5). Magnetic resonance imaging (MRI) demonstrated an obviously minimized tumorous lesion at the pancreas head (Figure 6). The mass lesion was visible as an iso-intensity signal in a T1-weighted image and as a faintly low-intensity signal in a T2-weighted image, while it was ill enhanced in an EOB image and the signal was heterogeneously repressed in a diffusion-weighted image. A subsequent CT, conducted one month after the FNAB, revealed further minimization of the pancreatic mass (Figure 1c,d). The images obtained in the next two months showed that the tumor had almost vanished. The tumor was no longer visible at the sixth month. During the post-diagnosis course, no medication was administered other than regularly taken hypotensive drugs. A written informed consent was obtained from the patient.

## 3. Discussion

Our English literature survey on PubMed using the keywords “inflammatory myofibroblastic tumor” with “pancreas” or “pancreatic” listed 167 citations. By excluding other inflammatory pancreatic pseudotumors (such as other mass-forming pancreatitis and autoimmune pancreatitis) and histology-unproven cases, 27 cases of histologically confirmed IMTs (positive for histologically proven myofibroblastic tissue) were listed (Table 1). The classical term “inflammatory pseudotumor (IPT)” often includes other etiologies, such as IgG4-related, autoimmune-related, and infection-causing inflammatory lesions [39]; therefore, we only listed the histologically proven pancreatic IMTs and not the IPTs. Including the current case, the mean age at diagnosis of all cases was 40.0 years, and a subtle predominance of male gender was evident (17 males and 11 females). The pancreatic mass lesions were mostly located in the pancreas head (20 in the head, two in the body, four in the tail, and two in the body and tail) and had a mean size of 4.7 cm. Abdominal pain or discomfort was the most frequent symptom (56%, 15/27), with jaundice the second most frequent (44%), followed by anorexia or weight loss (26%), and nausea or vomiting (15%). One case had stable disease, one case had a recurrence in the lung, and one case died of sepsis, but the other 22 (88%) cases did not show recurrence after resection or remission (Table 1). 

These data for pancreatic, IMTs differ greatly from the data of 730 IMT cases in 93 articles summarized by Nonaka et al. [1] who reported a younger onset (mean 29.6 years, range: 0–87 years), a nearly equal gender ratio, favored organs (commonest in lung, followed by the urinary bladder, mesentery, omentum, retroperitoneum, pelvis, gastrointestinal tract, and liver), larger size (mean 5.9 cm, range 0.4–36 cm), varied symptoms (strongly related to the location, but 19% were accompanied by systemic symptoms including fever, malaise, weight loss, and anemia), and different outcomes (local recurrence: 22%; metastasis: 3%; death from disease: 2%; and no recurrence: 67%). This trend became more apparent when comparing 59 cases of systemic IMTs with histological atypia [5], as these had younger onset (mean age: 13.2 years old, ≥20 years old: 29%), an even gender ratio (29 male and 30 female), large tumor size (mean: 7.8 cm), similar location (abdomen and pelvis: 64%, lung: 22%, head and neck: 8%, and extremity: 5%), and poorer outcomes (recurrence: 56%; metastasis: 10%; death from disease: 10%; and no recurrence: 42% in an average of 3.6 years of follow up). Thus, a wide range of variation exists in the nature of IMTs, and the adult pancreatic IMTs may have a relatively benign nature.

The biological marker of IMTs, including histological atypia, ganglion-like cells, TP53 expression, and aneuploidy pattern, have been correlated with more aggressive clinical behavior [40]. Coffin et al. also suggested that ALK (anaplastic lymphoma kinase) expression is another prognostic indicator of IMTs. The *ALK* (2p23) gene is activated by gene rearrangement in 50–70% of IMTs and *ALK* gene rearrangement is correlated with ALK expression, as determined by immunochemistry [41]. The absence of ALK expression in IMT was associated with a higher age of the patients [5]. All six of the observed metastases developed in 59 IMTs that were negative for ALK expression, and they developed before 20 years of age (mean age: 13.2 years), indicating a metastatic potential for ALK-negative IMTs in the younger subset [5]. The current case of a pancreatic IMT was in a patient of high age (82 years) and showed no histological atypia, ganglion-like cells, or ALK expression. Therefore, ALK expression as a clinical indicator in IMTs in older patients needs further evaluation.

IMTs show spontaneous regression in a minor fraction of patients, although the actual incidence is unclear due to surgical interventions and asymptomatic/undetected cases. To date, 13 cases of spontaneously regressed IMTs have been reported in various organs, but not in the pancreas (Table 2) [27,28,29,30,31,32,33,34,35,36,37,38]. As mentioned above, reported cases of pancreatic pseudotumors or those without proven myofibroblastic tissues were not listed [15,23]. Of the 13 cases, corticosteroids and/or NSAIDs were used in six cases and were effective in five cases. This phenomenon leads us to question the neoplastic nature of this tumor. IMTs often expand in size to invade multiple organs; therefore, when an accurate diagnosis can be made, conservative treatments should be recommended for older patients. 

An accurate diagnosis of pancreatic IMTs cannot be made by image examinations alone, as most cases mimic malignancies (Table 1) and no IMT serum markers are commercially available. EUS-FNA demonstrates a fairly high diagnostic ability (nearly 95% sensitivity and specificity) for solid malignant pancreatic lesions [42,43]. The use of thick core biopsy needles [44] and high-negative-pressure aspiration methods [45] has increased the acquisition rate for obtaining core tissue samples. This, in turn, has enabled the determination of the probable nature of the whole pancreatic mass and even the classification of intermediate inflammatory and neoplastic conditions, such as IMTs. In the present case, we performed a conventional EUS-FNA but used a 22-gauge Franseen needle, and our pathologist was able to diagnose IMT (Figure 4). Other similar conditions, such as other inflammatory pseudotumors (IgG4-related [39,46], autoimmune-related [47], and infection-related [39] masses) and spindle cell tumors (malignant fibrous histiocytoma [47], sarcomatoid anaplastic large cell lymphoma, spindle cell carcinoma, inflammatory leiomyosarcoma, and pleomorphic liposarcoma) [5], must be carefully ruled out by multiple immunohistochemical tests.

In conclusion, we have reported a rare case of pancreatic IMT demonstrating spontaneous remission. Our aim was to emphasize the importance of accurate diagnosis that includes histology obtained by EUS-FNA. Further accumulation of cases is needed to clarify the biological behavior of pancreatic IMTs.

## Figures and Tables

**Table 1:** Reported cases of inflammatory myofibroblastic tumor (IMT) of the pancreas (English literature).

No	Ref no.	Author	Year	Age (years old)	Gender	Location	Tumor Size (cm)	Symptoms	Pathological Examination (pathology)	Preoperative Diagnosis	Treatment	Course after Surgery or Remission	
												Follow-up Period	Status
1	26	Kroft	1995	42	F	Pb	7	abd. pain, weight loss, fatigue	FNAB (benign pancreatic tissue)	ND	resection	6 months	NER
2	6	Shankar	1998	8	F	Pbt	10.7	abd. pain	ND	sarcoma	resection	2 years	NER
3	7	Walsh	1998	35	M	Ph	5	jaundice, abd. pain, anorexia, weight loss	ND	pancreatic cancer	resection	6 years	lung recurrence
												3 years	NER
4	8	McClain	2000	11	F	Ph	3.4	jaundice, abd pain, weight loss	ND	malignancy	resection	ND	ND
5	9	Wreesman	2001	62	M	Ph	3	jaundice	ND	pancreatic cancer	resection	6 years	NER
6				56	M	Ph	ND	jaundice	ND	pancreatic cancer	resection	5 years	NER
7				50	M	Ph	5	jaundice	ND	pancreatic cancer	resection	4 years	NER
8				57	F	Ph	ND	jaundice	ND	pancreatic cancer	resection	3 years	NER
9				45	M	Ph	no mass	jaundice	ND	pancreatic cancer	resection	10 years	NER
10				32	F	Ph	2.5	abd. pain	ND	pancreatic cancer	resection	12 years	NER
11	10	Yamamoto	2002	55	M	Ph	1.5	none (incidental finding ^€^)	ND	pancreatic cancer	resection	28 months	NER
12	11	Esposito	2003	69	M	Pbt	ND	abd. pain	ND (no malignancy)	ND	resection	7 months	died of sepsis
13	12	Pungpapong	2004	70	M	Pt	3.8	none (incidental finding ^€^)	ND (no malignancy)	ND	resection	10 months	NER
14	13	Dulundu	2007	65	M	Pb	2	none (incidental finding ^€^)	ND	ND	resection	3 years	NER
15	14	Sim	2008	56	M	Pt	7	melena	ND	pancreatic cancer	resection	1.5 years	NER
16	15	Dagash	2009	13	F	Ph	3	jaundice, vomiting, weight loss	open bp ^¥^ (IPT)	ND	resection	6 years	NER
17				10	M	Ph	2.2	jaundice, abd. pain, anorexia	percutaneous bp (IPT)	IPT	predonisolone, cefuroxime	6 years	NER
18	16	Hassan	2010	19	M	Pt	8.2	abd. pain	ND	splenic rupture	resection	6 years	NER
19	17	Schutte	2010	44	F	Ph	6	abd. pain, nausea, weight loss	ND	malignancy	resection	1 year	SD
20	25	Lacoste	2012	56	M	Ph	ND	abd. pain	bp (not diagnostic)	possible malignancy	resection	ND	NER
21	18	Tomazic	2014	0	M	Ph	4	jaundice	US-FNAB ^£^ (not diagnostic)	possible malignancy	resection	3.5 years	NER
22	19	Panda	2015	32	F	Ph	4.8	jaundice, abd. pain	ND	pancreatic cancer	resection	2.5 years	NER
23	20	Zanchi	2015	13	F	Ph	2.5	jaundice, abd. pain, vomiting, anorexia	US-FNAB ^£^ (mesenchymal neoplasm)	mesenchymal neoplasm	resection	4 years	NER
24	21	Battal	2016	46	M	Ph	8	abd. pain	ND	ND	resection	ND	ND
25	22	Ding	2016	69	M	Ph	4	vomiting, anorexia	endoscopic bp. (fibrous lesion with inflammatory cells)	malignancy	resection	3 years	NER
26	23	Liu	2017	15	M	Pt	5	abd. pain	US-FNAB ^£^ (compatible with IMT)	tumor invading the transverse colon	Resection ^#^	3 years	NER
27	24	Berhe	2019	1	F	Ph	ND	ND	ND	IMT or chronic pancreatitis	resection	ND	ND
28		Current case	2019	82	F	Ph	5	abd. discomfort	IMT	IMT	None ^*^	9 months	NER
				Average	M:F	Ph:Pb:Pt:Pbt	Average						
				39.7	17:11	20:2:4:2	4.7						

M: male, F: female, ND: not described, NER: no evidence of recurrence, SD: stable disease, Ph: pancreas head, Pb: pancreas body, Pt: pancreas tail, Pbt: pancreas body to tail, IMT: inflammatory myofibroblastic tumor, IPT: inflammatory pseudotumor, abd pain: abdominal pain, ¥ bp: biopsy, US-FNAB £: abdominal ultrasound-guided fine needle aspiration biopsy, # tumor resection and segmental colorectomy, € incidentally detected by the health check images, * Current case showed spontaneous regression without medication.

**Table 2:** Reported cases of inflammatory fibroblastic tumor (IMT) with spontaneous and/or drug-used remission (English literature).

No.	Ref. no.	Author	Year	Age (years old)	Gender	Tumor Size (cm)	Location	Symptoms	Histological Examination	Treatment	Course after Remission
1	27	Przkora	2004	63	F	ND	retroperitoneum and mesentery	none	ND	predonisolone: 150 mg/day and diclofenac 50 mg × 2/day for 1 week	14 months, NER
2				22	M			abd. discomfort	ND	predonisolone: 150 mg/day and ibuprofen 400 mg × 2/day for 1 week	12 months, SD
3	29	Galindo	2008	28	M	ND	skull base	hearing loss, headache, otalgia	open bp	none	3 years, NER
4	30	Mattei	2008	13	M	ND	duodenum	ND	open bp	ketorolac	ND
5	31	Sugiyama	2008	72	M	ND	mediastinum	abd. discomfort, anorexia	US-guided bp	none	4 months, NER
6	32	Bilaceroglu	2009	21	F	6, 2	bilateral lung	abd. pain, vomiting, weight loss	needle bp, lung lobectomy	right lower lobectomy (none for left lung lesion)	1 year, NER
7	33	Fragoso	2011	14	M	diffuse involvement of segments IV–VIII	liver	none (anemia)	FNAB	antibiotics	6 years, NER
8	28	Shatzel	2012	28	F	5.5	mesentery	abd. pain	incomplete mass resection	prednisone 20 mg/day and celecoxib 200 mg/day for 2 weeks	3 months, shrunk to 4.2 cm
9	34	Calaway	2014	71	F	5	kidney	abd. pain, vomiting, fever	percutaneous FNAB (IMT)	none	ND, NER
10	35	Zhao	2014	49	M	15	retroperitoneum	abd. pain, vomiting	laparotomic incisional bp	none	3 months, NER
11				59	M	3.9	gastric wall	abd. distension, weight loss	endoscopic bp, laparotomic lymphadenectomy	none	1 year, NER
12	36	Markovic Vasiljkovic	2016	middle age	F	occupying entire pelvis	uterus	lumbago, weight loss, leg edema	open bp	none	5 years, NER
13	37	Yoshimura	2016	78	F	ND	cauda equina	pain and numbness in buttock	laminectomy, intraoperative bp	none	3 years, NER
14	38	Habib	2017	7	M	1.7	orbit	decreased visual acuity, color desaturation	orbital bp	corticosteroid (failure)	12 years, shrunk to 0.8 cm
15		Current case	2019	82	F	5	pancreas	abd. discomfort	EUS-FNAB	none	9 months, NER
		Average		43.4	(8:6)	5.6					

M: male, F: female, ND: not described, NER: no evidence of recurrence, SD: stable disease, EUS: endoscopic ultrasonography, FNAB: fine needle aspiration biopsy, bp: biopsy, IMT: inflammatory myofibroblastic tumor.

## Abbreviations

IMT	inflammatory myofibroblastic tumor
NSAIDS	non-steroidal anti-inflammatory drugs
CT	computed tomography
SMT	submucosal tumor
EUS	endoscopic ultrasonography
FNAB	fine needle aspiration biopsy
ASMA	anti-smooth muscle antibody
MRI	magnetic resonance imaging
IPT	inflammatory pseudotumor
*ALK*	anaplastic lymphoma kinase
